# Application of Receiver Operating Characteristic (ROC) Curves for Explosives Detection Using Different Sampling and Detection Techniques

**DOI:** 10.3390/s131216867

**Published:** 2013-12-06

**Authors:** Mimy Young, Wen Fan, Anna Raeva, Jose Almirall

**Affiliations:** Department of Chemistry and Biochemistry and International Forensic Research Institute, Florida International University, 11200 SW 8th St. OE 116A, Miami, FL 33199, USA; E-Mails: myoun002@fiu.edu (M.Y.); wfan001@fiu.edu (W.F.); aaraeva@gmail.com (A.R.)

**Keywords:** planar solid phase microextraction (PSPME), solid phase microextraction (SPME), ion mobility spectrometer (IMS), military explosive, smokeless powder, receiver operating characteristic curve (ROC)

## Abstract

Reported for the first time are receiver operating characteristic (ROC) curves constructed to describe the performance of a sorbent-coated disk, planar solid phase microextraction (PSPME) unit for non-contact sampling of a variety of volatiles. The PSPME is coupled to ion mobility spectrometers (IMSs) for the detection of volatile chemical markers associated with the presence of smokeless powders, model systems of explosives containing diphenylamine (DPA), 2,4-dinitrotoluene (2,4-DNT) and nitroglycerin (NG) as the target analytes. The performance of the PSPME-IMS was compared with the widely accepted solid-phase microextraction (SPME), coupled to a GC-MS. A set of optimized sampling conditions for different volume containers (1–45 L) with various sample amounts of explosives, were studied in replicates (*n* = 30) to determine the true positive rates (TPR) and false positive detection rates (FPR) for the different scenarios. These studies were obtained in order to construct the ROC curves for two IMS instruments (a bench-top and field-portable system) and a bench top GC-MS system in low and high clutter environments. Both static and dynamic PSPME sampling were studied in which 10–500 mg quantities of smokeless powders were detected within 10 min of static sampling and 1 min of dynamic sampling.

## 1. Introduction

Near perfect performance and excellent reliability are required of any instrumental system that detects chemical and biological threats in homeland security applications such as at transportation checkpoints. Ion mobility spectrometry (IMS) is the instrumental technique of choice for trace explosives detection in high throughput environments due to the high speed of analysis (∽1–10 s), excellent sensitivity (sub nanogram detection), low costs of acquisition and operation, robustness and portability. IMS detectors, however, have been reported to suffer from high false positive detection rates in high clutter environments, when particle swabbing is used as the sampling mode [1]. In response to this deficiency, different headspace sampling approaches that target the volatiles associated with the presence of explosives in containers have been developed. A novel sorbent coated disk coined planar solid phase microextraction (PSPME) system has been previously reported by our group for non-contact sampling of volatile organic compounds (VOCs) associated with explosives [2–4]. Unlike particle swabbing, PSPME targets the volatiles available in the headspace of large containers with fast preconcentration of the targets during the sampling. PSPME is reported to offer greater surface area and phase volume for fast sampling and larger capacity in comparison to the widely accepted solid phase microextraction (SPME) single fiber configuration. PSPME also offers the possibility of dynamic sampling of the air flow through the device with the assistance of a vacuum pump, allowing for large volume sampling, making it ideal for high throughput environments. Although the coupling of PSPME sampling to an IMS detector has been shown to perform well with respect to sensitivity and other performance metrics in the laboratory setting, an evaluation of the use of PSPME-IMS for explosives detection in real-world, high clutter settings had yet to be investigated.

The construction of receiver operating characteristic (ROC) curves are an efficient method to visualize the trade-offs for the performance of a particular technique or sensor system for a given set of sensor conditions. ROC curves were developed by the U.S. military to differentiate radar signals and noise [5,6] and this sensor performance evaluation tool has grown in popularity for use in medical diagnostic testing [7–10]. The increase in data analysis using ROC curves in non-clinical fields including psychiatry [11–13], explosives detection [14–17] and computer sciences [18–20] results from the ability to visualize the performance of dichotomous decisions. The Department of Defense (DoD) conducted the Chemical and Biological Sensor Standards Study [21] in which ROC curve studies for sensor devices were proposed based on a wide range of sensitivities and false positive rates. ROC curves can be constructed to display the instrument performance trade-offs of sensitivity and specificity from the true positive and false positive rates. From the DoD study, Cotte-Rodriguez and his colleagues constructed ROC curves for a portable mass spectrometer system to evaluate the real-time detection of toxic compounds [5]. Fraga *et al.* [22] also developed ROC curves for a portable IMS for vapor sampling of diesel fuels. In this study, the detection limit and performance of the instrument was determined under different defined scenarios.

This current study reports, for the first time, the development of ROC curves of the non-contact sampling of PSPME coupled with IMS detection including real-world sampling scenarios. ROC curves were constructed to evaluate the performance of two field-portable sampling systems and explosive detection systems with defined real-world scenarios for the detection of smokeless powders as a model for explosives. Smokeless powders are typically encountered in gunshot residues and have been used in improvised explosives [23,24]. Although smokeless powders are nonvolatile, volatile chemicals associated with the propellants and stabilizers can be used as target analytes for the detection of this class of explosives [25].

The performance of the PSPME-IMS technique was also compared with conventional fiber SPME extraction coupled to gas chromatography mass spectrometry (GC-MS) when calculating true-positive detection rates. Furthermore, several military-grade explosives were also sampled to evaluate the performance of the PSPME-IMS as a non-contact vapor sampling technique for the detection of military explosives. Table 1 lists the targeted volatile chemicals emitted from smokeless powders as well as the military explosives that were investigated in this study including their vapor pressures and reduced mobilities (K_0_).

## 2. Experimental Section

### 2.1. Instrumentation

The true positive rate (TPR) studies were conducted with two different techniques: PSPME-IMS (bench-top instrument and portable instrument) and SPME-GC-MS. The bench top IMS system used was an IONSCAN 400B (Smiths Detection, Mississauga, ON, Canada) which was used in both negative and positive polarity with nicotinamide and hexachloroethane dopants, as recommended by the manufacturer. A Morpho Detection Hardened MobileTrace was used as the portable IMS system and operated in the Explosives Particle Mode with dichloromethane (VICI Metronics, Inc., Poulsbo, WA, USA) and ammonia (Real Sensors, Inc., Hayward, CA, USA) dopants. For both instruments, the instrumental parameters were kept at the manufacturer's default parameters. The parameters for the benchtop IMS used the drift tube temperatures of 115 °C and 235 °C in the negative and positive polarity, respectively. The portable IMS system allowed for detection of analytes in both polarities, using the explosives particle mode setting with a drift tube temperature of 162 °C. Alarms for compounds not present in the library were added and the parameters used were similar to the alarms in the library. The alarm thresholds for the analytes of interest were adjusted to the minimum alarm threshold for true positive and false positive rate studies but a full listing of the alarm thresholds for each analyte in both IMS systems is presented in Table 2.

The GC-MS studies were performed using a Varian (Palo Alto, CA, USA) CP 3800 gas chromatograph coupled to a Saturn 2000 ion trap mass spectrometer and equipped with an CP 8400 autosampler (Varian Inc., Walnut Creek, CA, USA). The sample was introduced to the GC with an inlet temperature of 180 °C (split ratio 5:1) and analyzed using a 30 m × 0.25 mm ID × 0.25 µm DB-5MS UI (Agilent Technologies, Inc., Santa Clara, CA, USA) with a constant flow rate of Helium at 2.0 mL•min^−1^. The method length was 29.3 min, in which the GC oven started at a temperature of 40 °C and held for 1 min, followed a ramp to 200 °C at 15 °C·min^−1^, then held for 1 min, another ramp to 240 °C at 15 °C·min^−1^, held for 6.5 min, a third ramp to 270 °C at 25 °C·min^−1^, then a final ramp to 280 °C at 5 °C·min^−1^, held for 4 min. The transfer line to the ion trap was set to 280 °C and the ion trap was maintained at 180 °C. Each compound of interest was identified by the retention of their pure standards and identifying the resulting peak using the NIST mass spectral library.

### 2.2. Materials and Methods

A planar solid phase microextraction (PSPME) device is an acid-cured glass fiber filter (Fisher Scientific, Pittsburgh, PA, USA) that is spin-coated (Laurell Technologies Co., North Wales, PA, USA) with a sol-gel solution as previously described [2]. The size of the PSPME disk was modified in order to fit the geometry of the MobileTrace thermal desorption system. The PSPME for the bench-top instrument was introduced with the assistance of a Teflon holder (Field Forensics, St. Petersburg, FL, USA) without further modification.

The different containers of varying materials and volumes sizes were used in this study. This includes metal quart and gallon cans (All-American Containers, Miami, FL, USA) of 0.94 and 3.8 L, respectively, as well as polypropylene plastic containers of 45 L (15.625 × 13.125 × 13.25 inches in dimension). Prior to use, the metal cans were baked at 100 °C for over 24 h in order to remove residual volatiles from the manufacturing process and any background volatiles adsorbed on the surfaces of the cans. The plastic containers were used without further modification but blank samples revealed no interfering compounds. 10–500 mg of smokeless powders (Alliant Powder Unique (AU), Radford, VA, USA), IMR Powder Co. 4198 (Shawnee Mission, KS, USA) were placed directly in the containers or in a Petri-dish (Fisher Scientific) within the containers and sealed. All headspace studies were performed at room ambient temperature (∽23 °C). For equilibrium studies, static headspace sampling of 10 min was performed at different times after sealing the containers (0–72 h) and sampled in triplicate. The observed signals were plotted against the elapsed times to determine the headspace equilibrium within a given volume. Headspace PSPME extractions were performed statically, in which the PSPME disk was exposed to the headspace of the closed system for a given amount of time, as well as dynamically with the assistance of air flow using the Barringer remote DC sampler at 0.17 L s^−1^ for no more than 1 min. Dynamic extractions were performed by lifting the lid of the containers and sampling with the lid on top of the sampling device in order to contain the vapors. Thirty replicates were performed for the TPR studies for each different defined scenario. ROC curves were constructed using a commercially available statistical analysis software (JMP v. 10).

For SPME-GC-MS analysis, polydimethylsiloxane (PDMS) and PDMS/divinylbenzene (DVB) SPME fibers (Supelco, Sigma-Aldrich Corp., St. Louis, MO, USA) were both used for the sampling and headspace preconcentration and resulted in very similar extraction efficiency for the target volatile compounds. However, the use of the combined PDMS/DVB SPME fibers resulted in improved sensitivity over the single sorbent fibers. A hole was punctured on the top of the lid of each metal can container and sealed with a red rubber sleeve stopper that was used for introduction of the SPME fiber into the sealed system for headspace extraction. After exposing the fiber for 10–60 min, it was retracted and then analyzed by using GC-MS.

Military explosives including cyclotrimethylenetrinitramine (RDX), pentaerythritol tetranitrate (PETN), erythritol tetranitrate (ETN), nitroglycerin (NG) and ethylene glycol dinitrate (EGDN) were synthesized and characterized by the Tyndall Air Force Base (Panama City, FL, USA). All handling and disposing of the explosives were carried out by the explosives team. The solid explosives were weighed to 500 mg and placed in a glass watch glass or small plastic container and then placed in the 3–4 L plastic container (Sterilite Corporation, Townsend, MA, USA). For the liquid explosives (NG & EGDN), the plastic bottle originally containing the explosive was directly placed in the 3–4 L plastic container with the removal of the bottle lid. These explosives were given 0–2 h of equilibrium time to allow for headspace buildup of the target volatiles. Empty explosive wrappers (TNT and C4) were placed in a plastic bag for 1–24 h for headspace buildup. Sampling was performed by opening the plastic bag and placing the nose of the air sampler at the opening of the plastic bag. Detection of these explosives was performed with the portable IMS using a maximum of 12 replicate measurements.

## 3. Results and Discussion

### 3.1. Optimized Parameters for ROC Curves

Equilibrium studies were performed in different containers to determine the amount of time required to achieve optimum detection of the target analytes. For all the containers, 24 h was sufficient for the VOCs to establish equilibrium; however, detection of the analytes of interest can be achieved within 1 h of equilibrium or buildup of the target volatiles. The optimized conditions for the containers of varying volumes are summarized in Table 3. For the three different volume sizes used in this study, the optimum static sampling time was determined to be 10 min for SPME-GC-MS and PSPME-IMS and 1 min for dynamic sampling using PSPME-IMS, with sample sizes ranging from 10–50 mg for both smokeless powders. Experiments using SPME-GC-MS were limited to static headspace extractions for the sampling of quart and gallon can containers and did not include sampling of the large plastic containers due to the impractical nature of sampling large volumes with static SPME sampling (requiring 30 min for the detection of analytes of interest).

Nitroglycerin (NG) and 2,4-dinitrotoluene (2,4-DNT) were observed to be the most abundant analytes in the headspace of smokeless powders, resulting in detection within 10 min of static extraction with a minimum of 10 mg of smokeless powder present in different containers. Dynamic extractions of 1 min were sufficient for detection of NG and 2,4-DNT from 10 mg of smokeless powders. The sensitivity for detection of DPA was greater in the bench top IMS, resulting in detection of DPA for all the different defined volumes. On the other hand, DPA detection required a minimum of 50 mg of AU smokeless powder in quart and gallon cans for the portable IMS detection system. Detection of DPA was difficult for the large volume containers in which DPA was not detected after 10 min of static sampling but detected within 1 min of a dynamic extraction.

NG, DPA and 2,4-DNT were successfully detected after a 10 min extraction for all the smokeless powders with as low as 10 mg of smokeless powder in both quart and gallon cans when SPME-GC-MS was used. However, the sampling of large volume containers required longer extraction times (30 min) for detection using the SPME-GC-MS, reducing the true positive detection rates (TPR) for large containers.

### 3.2. True Positives Rate Studies of Smokeless Powders

#### 3.2.1. PSPME Coupled with IMS

**Figure 1.**
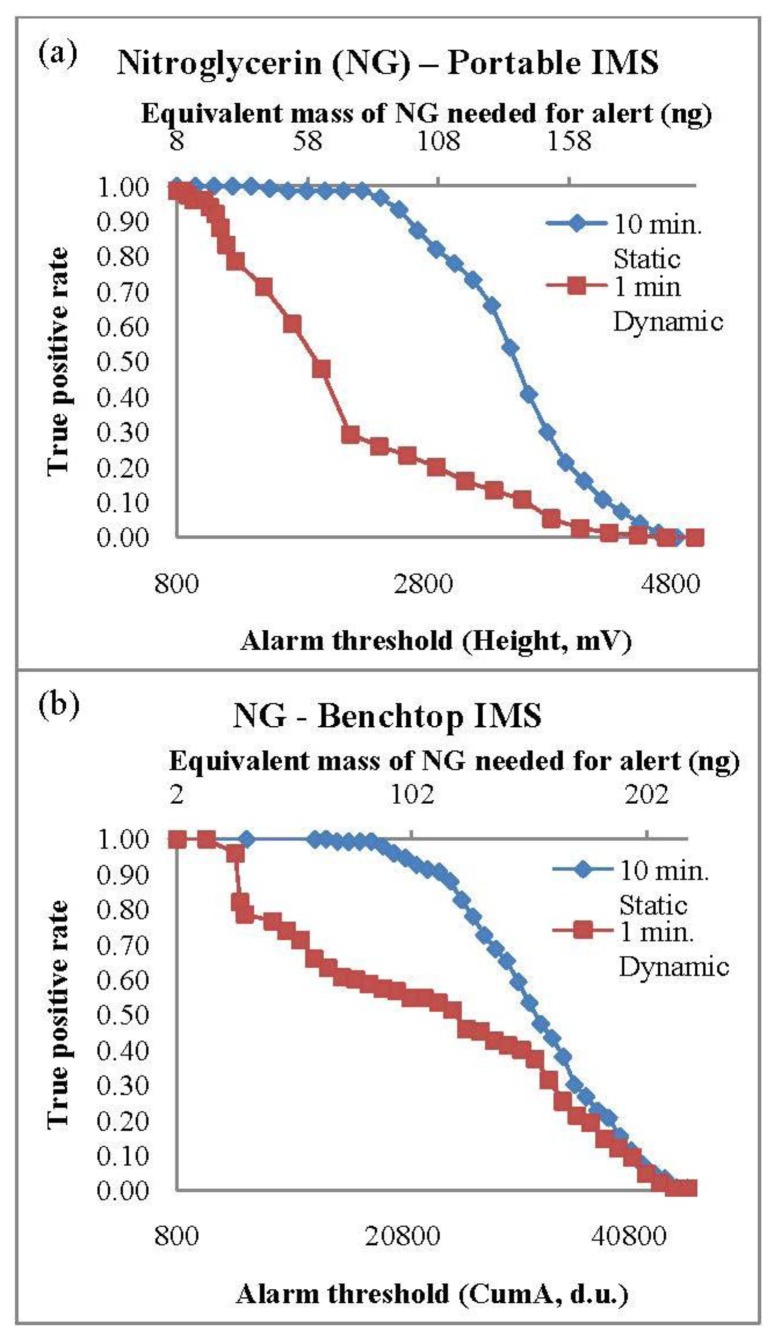
True positive rates for the (**a**) portable and (**b**) benchtop IMS systems. Comparison of true positive rates for the two extraction methods are shown with varying alarm threshold.

PSPME coupled with both portable and bench-top IMS systems resulted in excellent detection performance for both 2,4-DNT and NG. The TPR values were calculated based on the fraction of containers containing smokeless powders that resulted with a maximum signal (in height, mV) greater than the predetermined alarm threshold value. The TPR curves with respect to the alarm threshold set on the instrument for NG for both IMS systems are shown in Figure 1. The TPR decreases with increased alarm threshold with a TPR of 1.0 observed with a minimum detection equivalent to 8 ng and 2 ng of NG in the portable and bench-top IMS, respectively. A complete list of the TPRs results for the different scenarios for the three analytes of interest with the minimum alarm threshold are shown in Table 4. Static extractions for both IMS instruments showed a greater TPR values in comparison to dynamic extractions, nevertheless, the TPRs for 2,4-DNT and NG in the two systems for all the different set conditions were greater than 0.80. Detection of DPA was not very successful, resulting with the highest TPR of 0.82 for static extractions and 0.53 for dynamic extractions in the bench top IMS system. The highest TPRs for the DPA detection were 0.58 and 0.47 from static and dynamic extractions, respectively. Since DPA is a stabilizer [23,31] in the smokeless powders, the presence of other chemicals such as the explosive NG is required for a positive alarm of low explosives. The presence of DPA can use used as a confirmation for the detection of smokeless powders.

#### 3.2.2. SPME Coupled with GC-MS

**Figure 2.**
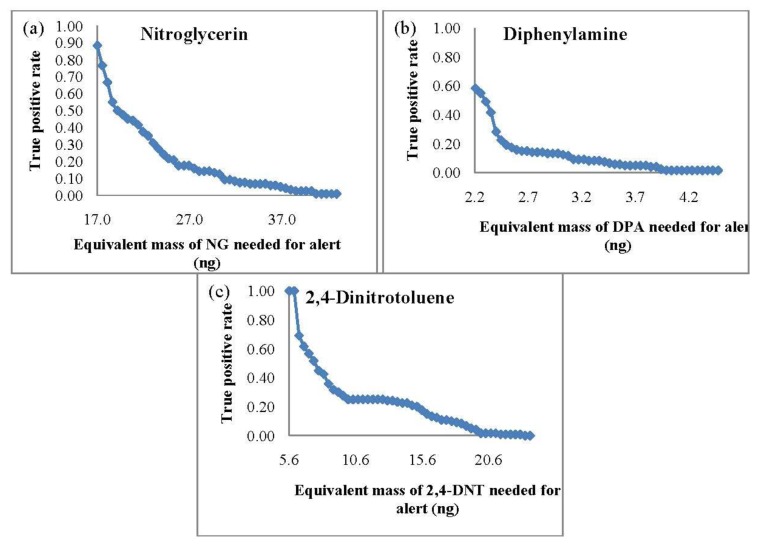
SPME-GC-MS true positive rates with varying equivalent mass threshold for (**a**) NG, (**b**) DPA and (**c**) 2,4-DNT.

Since SPME is limited to static extractions, the same extraction time of 10 min was repeated for the SPME-GC-MS TPR studies. The TPR curves based on the equivalent mass detection (from the integrated area) of the target analytes are shown in Figure 2.

In comparison to the PSPME-IMS studies, SPME-GC-MS led to poorer sensitivity with a TPR of 0.88 and a minimum detection of 17 ng for NG and a TPR of 1.0 with equivalent mass detection of 6 ng for 2,4-DNT. Detection of DPA was slightly better with a TPR of 0.58 with detection equivalent to 2 ng of DPA. These results show that the SPME-GC-MS system is sensitive for 2,4-DNT and DPA; however, detection of NG is much more sensitive using PSPME coupled with commercial IMS.

### 3.3. True Positive Rate Studies of Military Explosives

For the military explosives study, 1 min dynamic extractions followed by IMS detection using the portable system was performed with a maximum of 12 replicates for the different explosives available. Most of the military explosives were not detected in the portable IMS system due to their low vapor pressure. A true positive rate of 0 was determined for the vapor sampling of 500 mg of ETN, PETN and RDX in a 3–4 L plastic container. Volatile explosives such as NG and EGDN resulted in excellent detection performance with a TPR of 1.0 with EDGN and NG producing an alarm in the IMS for the NITRO alarm set. The high volatility of these explosives resulted in relatively large amounts of the volatiles to be preconcentrated onto the PSPME device. Additionally, wrappers of explosives were sampled resulting in a TPR of 0.60 for TNT, in which the alarm was based on the detection of 2,4-DNT, the primary volatile organic compound associated with TNT [32] from the headspace of the wrappers. The C4 explosives are primarily composed of RDX, thus, resulting in no detection of explosives from the wrappers. Detection of the 2,3-dimethyl-2,3-dinitrobutane (DMNB) taggant present in plastic explosives was possible; however, when a lower drift tube temperature was used as previously reported [33].

### 3.4. False Positive Rate Studies

False positive rates (FPR) were determined in replicates under the same conditions as the TPR studies, but in the absence of explosives. These measurements were collected in the laboratory (relatively low clutter) as well as an outside loading dock area (high particle clutter) in order to simulate real-world scenarios and observe typical backgrounds from cluttered shipping environments. A total of 10 replicates measurements were collected for each defined parameter. Similarly, the FPR values were calculated based on the fraction of the containers that did not contain explosives but resulted in a maximum signal (in Height, mV).

From the 10 replicates, the portable IMS system resulted in no false positives. Since the alarm threshold was decreased for the sensitive detector, the benchtop IMS resulted with a FPR of 0.06. By increasing the minimum alarm threshold of the analytes of interest will still result with a TPR of 1.0 (>800 d.u.) and the FPR can be decreased to 0.

**Figure 3.**
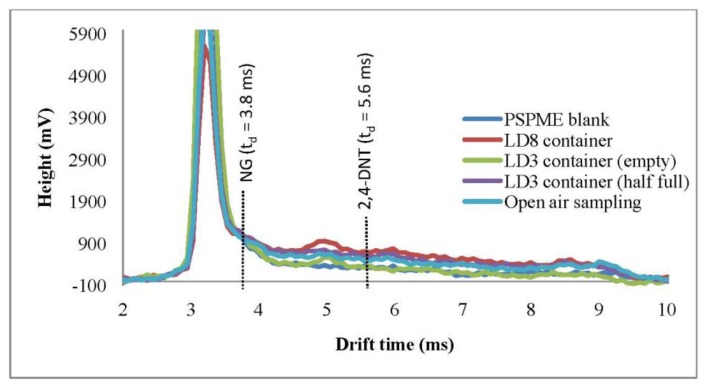
Plasmagrams for dynamic PSPME sampling (1 min.) in cluttered environments from a local shipping facility. Sampling was performed in LD3 (4,500 L) containers and LD8 (6,880 L) containers as well as open air sampling of the location with indication of detection windows for NG and 2,4-DNT (drift time (t_d_) of 3.8 ms and 5.6 ms, respectively).

FPR studies were also performed in a local commercial shipping facility in which several different containers were sampled as well as the headspace of the open area with only the portable IMS used in this part of the study. One-min dynamic sampling with IMS detection was performed in open areas as well as inside LD3 containers. Plasmagrams of the negative mode for the portable IMS shows a signal (t_d_ = 8.6 ms) reflecting the presence of background volatiles in the headspace of the LD3 containers (Figure 3), however, none of the signals obtained from the background interfered with the analytes of interest. Moreover, from a total of 32 background samples that were sampled by PSPME-IMS in this highly cluttered environment, none caused a false positive alarm.

### 3.5. Receiver Operation Characteristic Curves for PSPME-IMS Systems

**Figure 4.**
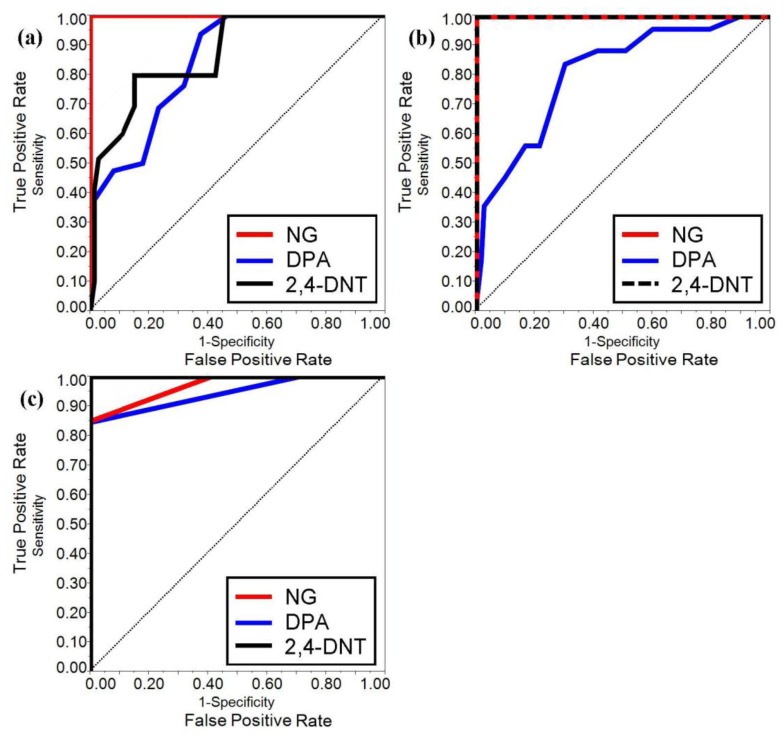
ROC curves for the portable PSPME-IMS (**a**), benchtop PSPME-IMS (**b**) and laboratory based SPME-GC-MS (**c**). These ROC curves were constructed using JMP software from 360 samples (140 samples for SPME-GC-MS) including all defined scenarios.

The ROC curves were developed for both benchtop and portable IMS systems when coupled with PSPME devices from the all the defined scenarios and replicates to determine the overall performance for detection of the target analytes for the different sampling and detection techniques. From a total of 360 samples for all the different replicates and the different defined scenarios, the ROC curves for the two instruments were constructed for the three target analytes using JMP (version 10) software (SAS Institute Inc., Cary, NC, USA). The results for the different scenarios were used to determine the sensitivity (TPR) and specificity (1-FPR) trade-offs for the target analytes as shown in Figure 4a and b for the portable and benchtop IMS, respectively. The area under the curves (AUC) of the benchtop IMS were greater for analyte 2,4-DNT than the portable IMS (1.0 and 0.87, respectively) with similar performance for detection of DPA (AUC of 0.81 and 85 for the benchtop and portable IMS, respectively) and a perfect ROC curve (AUC = 1.0) was determined for NG in both detector systems. The results indicate that the portable PSPME-IMS system achieved similar detection performance for DPA as the benchtop PSPME-IMS instrument because of similar sensitivity and limits of detection for the two instruments; however, increased positive alarms from the portable PSPME-IMS in the plastic containers (TPR = 0.27) in comparison to the benchtop PSPME-IMS instrument (TPR = 0.12) under the same scenario showed slightly improved performance for the portable PSPME-IMS. The overall performance of the two PSPME-IMS systems showed excellent performance, with similar or greater performance of the benchtop PSPME-IMS in comparison to the portable PSPME-IMS system.

ROC curves were also constructed for the laboratory based SPME-GC-MS technique as shown in Figure 4c, which include only static extractions and excluding scenarios involving plastic containers resulting with a total of 140 samples. The sampling and detection technique resulted with better performance compared to the PSPME-IMS system with a perfect ROC curve (AUC = 1.0) for 2,4-DNT and excellent performance for detection of NG and DPA with an AUC of 0.97 and 0.94, respectively. The poor sensitivity of NG in the SPME-GC-MS technique resulted in poorer performance in comparison to IMS detection systems. Overall, the SPME-GC-MS resulted with excellent performance for all the analytes of interest under the defined scenarios as expected for a sensitive, laboratory based instrument; however, PSPME-IMS offers similar non-contact sampling and detection performance to a well-established technique with the added advantage of fast detection in the field.

## 4. Conclusions

The performance of the planar solid phase microextraction (PSPME) non-contact sampler/extraction device coupled to COTS ion mobility spectrometers (IMSs) to detect the presence of explosives was evaluated through the development of receiver operating characteristic (ROC) curves. A total of 360 replicate measurements were collected for different scenarios varying container volume (0.94–45 L) and amount of smokeless powders concealed within the container (10–500 mg). True positive rate (TPR) analysis suggested the optimum alarm threshold and detection limits for each individual compound.

ROC curves are found useful to illustrate the detector system performance in terms of true and false positive probabilities. The portable IMS resulted in slightly reduced performance; however, the instrument performed well with high sensitivity for NG (AUC = 1.0) and 2,4-DNT (AUC = 0.87). DPA resulted with a lower AUC of 0.85 due to large amount of false negatives. The benchtop IMS resulted in improved sensitivity resulting with an area under the curve (AUC) of 1.0 for NG and 2,4-DNT and similar performance for DPA with an AUC of 0.81. Even though poor detection was observed for DPA for both IMS instruments, the presence of NG from the same smokeless powders was sufficient for a positive alarm, suggesting the presence of an explosive. Moreover, although SPME-GC-MS resulted with better performance with an AUC greater than 0.9 under the defined scenarios, SPME requires a minimum of 10 min static headspace sampling and the laboratory-based GC-MS requires long analysis time which is impractical in high throughput locations. This study illustrates how well the preconcentration power of the PSPME is able to perform in brief (∽1 min.) static and dynamic extractions with high sensitivity and high specificity and can be coupled to the 15,000 IMS instruments currently deployed at security checkpoints throughout the US without further modification. However, detection of explosives with low vapor pressure by non-contact sampling was not successful, resulting in little to no detection for ETN, PETN, RDX, TNT and C4. Further optimization studies will be investigated in order to construct ROC curve studies based on the volatile organic compounds associated with these low vapor explosives. Overall, the PSPME-IMS technique provides less false positive results for non-contact vapor sampling, cutting the cost and providing an effective sampling and detection needed in high-throughput scenarios with excellent potential to be a used as a sensor system for the detection of volatile chemicals associated with explosives.

## Figures and Tables

**Table 1.** Volatile compounds detected in smokeless powders. Vapor pressures are from references [26–30]. K_0_ values as programmed in the Smiths Detection IMS instrument.

**Name**	**Chemical Structure**	**Vapor Pressure, Torr (25°C)**	**Reduced Mobility, K_0_(cm^2^•V^−1^•s^−1^)**
2,4-Dinitrotoluene (2,4-DNT)	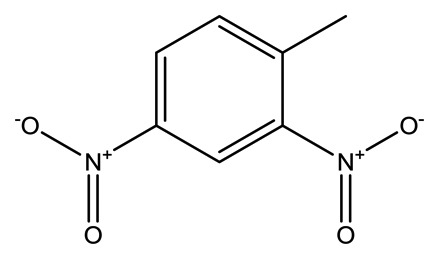	2.1 × 10^−4^	1.5660
Diphenylamine (DPA)	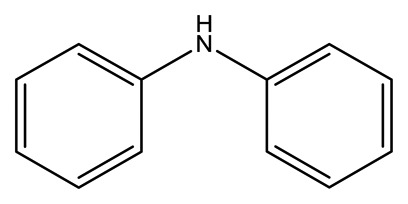	2.7 × 10^−3^	1.6082
Ethylene glycol dinitrate (EGDN)	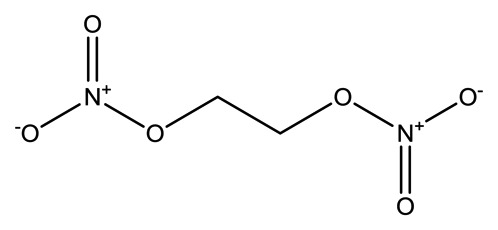	7.7 × 10^−2^	1.528
Nitroglycerin (NG)	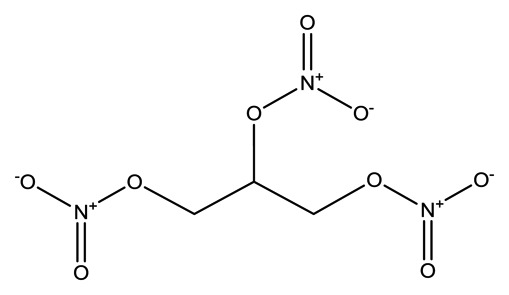	1.8 × 10^−3^	1.2720 (NG-N), 1.3385 (NG-C)
Erythritol tetranitrate (ETN)	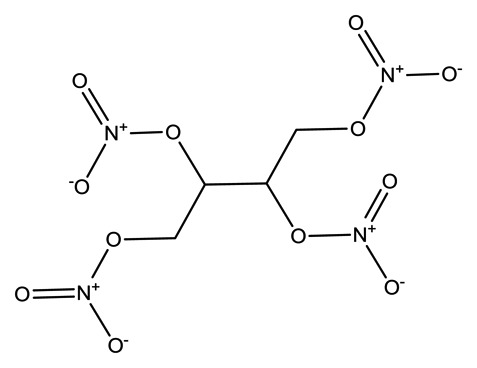	2.4 × 10^−5^	1.8842
Pentaerythritol tetranitrate (PETN)	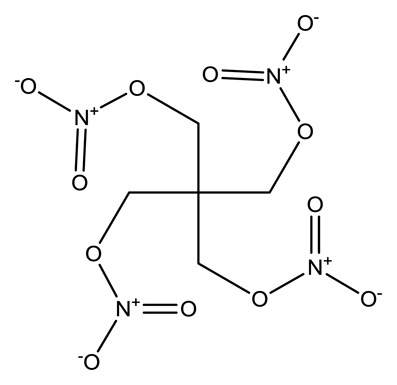	4.0 × 10^−8^	1.0999
Cyclotrimethyl- enetrinitramine (RDX)	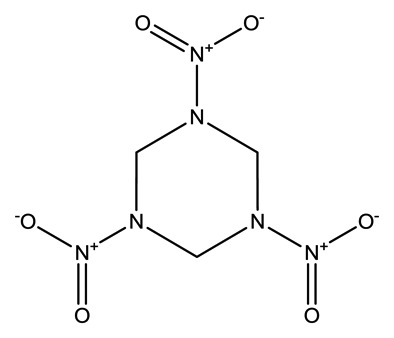	1.7 × 10^−8^	1.3129
2,4,6- Trinitrotoluene (TNT)	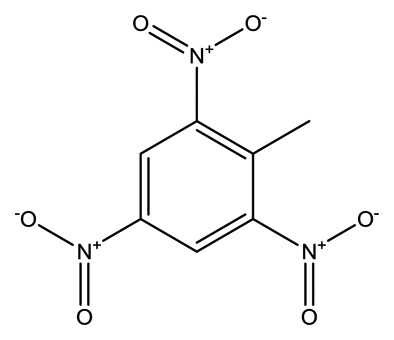	8.8 × 10^−6^	1.4488

**Table 2.** Alarm threshold for analytes of interest for benchtop and portable IMS systems. Military explosives were only detected using the portable IMS, thus, parameters for these analytes are only shown for the portable IMS.

**Alarm**	**Smiths Detection IONSCAN**				**Morpho Detection Hardened** **MobileTrace**		
	**Reduced mobility (K_0_)**	**Variability (µs)**	**Amplitude threshold (d.u.)**	**Full width half max (µs)**	**Drift time (ms)**	**Variability (ms)**	**Method of detection**
(+) DPA	1.6082	50	30	327	6.080	0.040	Height (100)
(−) 2,4-DNT	1.5660	50	30	253	5.548	0.040	Height (300)
(−) NG-N (−) NG-C	1.2720 1.3385	45 45	25 50	335 305	3.833	0.035	Height (700)
(−) ETN					4.672	0.040	Height (100)
(−) PETN					7.991	0.040	Height (500)
(−) RDX					6.333	0.040	Slope (1000)
(−) TNT					6.076	0.040	Height (300)

**Table 3.** Detection of analytes of interest (NG and DPA from All Unique smokeless powder; 2,4-DNT from IMR 4198 smokeless powder) for different sampling parameters.

**Container**	**Equilibrium** **Time (h)**	**Extraction**	**Sample** **Size (mg)**	**Sampling** **Time (min)**	**Instruments & Analytes Detected**		
					**PSPME-IMS** **(Portable)**	**PSPME-IMS** **(Bench Top)**	**SPME-GC-MS** **(Bench Top)**
Quart can (0.94 L)	24	static	10	10	NG, 2,4-DNT	NG, DPA, 2,4-DNT	NG, 2,4-DNT
			50	10	NG, DPA, 2,4-DNT	NG, DPA, 2,4-DNT	NG, DPA, 2,4-DNT
		dynamic	10	1	NG, 2,4-DNT	NG, DPA, 2,4-DNT	n/a
			50	1	NG, DPA, 2,4-DNT	NG, DPA, 2,4-DNT	
Gallon can (3.8 L)	24	static	10	10	NG, 2,4-DNT	NG, DPA, 2,4-DNT	NG, 2,4-DNT
			50	10	NG, DPA, 2,4-DNT	NG, DPA, 2,4-DNT	NG, DPA, 2,4-DNT
		dynamic	10	1	NG, 2,4-DNT	NG, 2,4-DNT	n/a
			50	1	NG, DPA, 2,4-DNT	NG, DPA, 2,4-DNT	
Plastic container (45L)	24–48	static	500	10	NG, 2,4-DNT	NG, 2,4-DNT	
		dynamic	500	1	NG, DPA, 2,4-DNT	NG, DPA, 2,4-DNT	

**Table 4.** True positive rates for smokeless powders in different containers (1–45 L) for bench top and portable IMS systems with 60 replicates.

		**Benchtop IMS**		**Portable IMS**	

**Container Volume (L)**	**Analyte**	**Static**	**Dynamic**	**Static**	**Dynamic**

0.94	NG	1.0	1.0	1.0	1.0
	DPA	0.70	0.37	0.58	0.15
	2,4-DNT	1.0	1.0	1.0	0.87

3.8	NG	1.0	1.0	1.0	1.0
	DPA	0.82	0.53	0.25	0.08
	2,4-DNT	1.0	1.0	0.98	0.98

45 *	NG	1.0	1.0	1.0	1.0
	DPA	0.0	0.23	0.07	0.47
	2,4-DNT	1.0	1.0	0.80	0.98

* denotes *n* = 30.
